# Correction: Osteoporotic hip fracture—Comorbidities and factors associated with in-hospital mortality in the elderly: A nine-year cohort study in Brazil

**DOI:** 10.1371/journal.pone.0294101

**Published:** 2023-11-02

**Authors:** Viviane Cristina Uliana Peterle, Maria Rita Carvalho Garbi Novaes, Paulo Emiliano Bezerra Junior, João Carlos Geber Júnior, Rodrigo Tinôco Magalhães Cavalcante, Jurandi Barrozo da Silva Junior, Ray Costa Portela, Ana Patricia de Paula

The third, fourth, and sixth authors’ surnames are written incorrectly. The correct format is: Paulo Emiliano Bezerra-Júnior, João Carlos Geber-Júnior, Jurandi Barrozo da Silva-Júnior.

The correct citation is: Peterle VCU, Novaes MRCG, Bezerra-Júnior PE, Geber-Júnior JC, Magalhães Cavalcante RT, da Silva-Júnior JB, et al. (2022) Osteoporotic hip fracture-Comorbidities and factors associated with in-hospital mortality in the elderly: A nine-year cohort study in Brazil. PloS ONE 17(8): e0272006. https://doi.org/10.1371/journal.pone.0272006

There is an error in affiliation 3 for author João Carlos Geber-Júnior. The correct affiliation is: Department of Internal Medicine, Hospital das Clínicas, Faculdade de Medicina, Universidade de São Paulo, São Paulo, Brazil, BR.
