# Supplementary material for: Osteoporotic hip fracture—Comorbidities and factors associated with in-hospital mortality in the elderly: A nine-year cohort study in Brazil
Source: PLoS One. 2022 Aug 12;17(8):e0272006. doi: 10.1371/journal.pone.0272006 (PMC9374234; doi:10.1371/journal.pone.0272006)
Supplement: S1 File — (DOCX) [file pone.0272006.s002.docx]

S2. Statistical Reporting

1. Methodology

The statistical analysis consisted of obtaining frequencies of the variables of interest, obtaining the incidences with the respective confidence intervals of the characteristics of the variables under study associated with the mortality outcome, and bivariate analysis, using as a measure of effect the prevalence ratio and their respective confidence intervals; Poisson regression analysis with robust variance.

In the present study, the mortality outcome was considered a dependent variable. The independent variables collected were (a) demographic: sex, age (60 to 79, 80 to 89, > 90 years); (b) factors associated with clinical conditions and comorbidities: hemoglobin (≤ 10 g/dL, > 10 g/dL), systemic arterial hypertension (SAH) (no, yes), diabetes mellitus (DM) (no, yes), neurological disorders (no, yes), chronic obstructive pulmonary disease (COPD) (no, yes), surgical risk (low to moderate, high), femur fracture (intracapsular, extracapsular); and (c) in-hospital variables: type of surgery (did not undergo surgery, osteosynthesis, arthroplasty), respiratory infection (no, yes), urinary tract infection (no, yes), another type of infection (no, yes) and (none, one, two or more), pulmonary thromboembolism (PTE) (no, yes), intensive care unit (ICU) stay in days (≤ 3, > 3).

To test the effect of independent variables on the mortality outcome, Poisson regression models with robust variance were used, obeying the hierarchical model proposed: demographic factors make up the first stage of analysis. The factors related to the preoperative variables, the second and third stages of analysis, are formed by the variables related to the postoperative period.

The Poisson regression model with robust variance was chosen because it provides a better estimate of incidence ratios, which represent the measures of effect for prospective studies, such as the relative risk (RR), in a more significant manner. The analysis took place in two phases, bivariate and hierarchical multiple regression analysis, and their RRs and respective 95% CI were calculated.

The association between each independent variable and the mortality outcome was verified in the bivariate analysis. Subsequently, multiple analysis was performed. In this analysis, variables with p < 0.10 were considered adjustment factors for subsequent analysis within each hierarchical level [1,2].

Multicollinearity between the independent variables was evaluated. It was considered as a limit of the presence of multicollinearity if the tolerance indicator assumes values lower than 0.403.

P < 0.05 was considered significant. The analyses were conducted using the SAS 9.4 software.

Mortality outcome according to the variables studied

| **Variables** | | **Patients**  **(n = 402)** | **Percentage**  **(%)** | **Mortality (%)** | **95 % IC** |
| --- | --- | --- | --- | --- | --- |
| **Sex** | Women | 260 | 64.68 | 17.31 | 12.69–21.93 |
|  | Men | 142 | 35.32 | 20.42 | 13.76–27.08 |
| **Age** | 60 - 69 | 78 | 19.40 | 5.13 | 0.21–10.04 |
|  | 70 - 79 | 130 | 32.34 | 8.46 | 3.66–13.27 |
|  | 80 - 89 | 142 | 35.32 | 25.35 | 18.17–32.54 |
|  | > 90 | 52 | 12.94 | 44.23 | 30.67–57.79 |
| **Hemoglobin** | ≤ 10 | 87 | 21.64 | 28.74 | 19.19–38.28 |
|  | > 10 | 315 | 78.36 | 15.56 | 11.54–19.57 |
| **Comorbidities** | No | 49 | 12.19 | 8.16 | 0.46–15.86 |
|  | Yes | 353 | 87.81 | 19.83 | 15.65–24.01 |
| **SAH** | No | 97 | 24.13 | 12.37 | 5.79–18.95 |
|  | Yes | 305 | 75.87 | 20.33 | 15.79–24.86 |
| **DM** | No | 263 | 65.42 | 16.35 | 11.86–20.85 |
|  | Yes | 139 | 34.58 | 22.30 | 15.35–29.25 |
| **Neurological disorders** | No | 296 | 73.63 | 15.88 | 11.70–20.06 |
|  | Yes | 106 | 26.37 | 25.47 | 17.14–33.80 |
| **COPD** | No | 371 | 92.29 | 15.90 | 12.17–19.64 |
|  | Yes | 31 | 7.71 | 48.39 | 30.72–66.05 |
| **Surgical risk** | Low to moderate | 268 | 66.67 | 8.58 | 5.21–11.95 |
|  | High | 134 | 33.33 | 38.06 | 29.80–46.32 |
| **Femur fracture** | Intracapsular | 218 | 54.23 | 18.81 | 13.60–24.02 |
|  | Extracapsular | 184 | 45.77 | 17.93 | 12.37–23.50 |
| **Type of surgery** | Non-surgical | 92 | 22.89 | 38.04 | 28.08–48.01 |
|  | Osteosynthesis | 173 | 43.03 | 13.87 | 8.70–19.05 |
|  | Arthroplasty | 137 | 34.08 | 10.95 | 5.70–16.20 |
| **Infection** | No | 267 | 66.42 | 2.25 | 0.46–4.03 |
|  | Yes | 135 | 33.58 | 50.37 | 41.90–58.84 |
| **Respiratory infection** | No | 316 | 66.42 | 4.43 | 2.15–6.71 |
|  | Yes | 86 | 21,39 | 69.77 | 60.02–79.51 |
| **Urinary tract infection** | No | 338 | 84.08 | 14.20 | 10.46–17.94 |
|  | Yes | 64 | 15.92 | 40.62 | 28.54–52.71 |
| **Other infection** | No | 386 | 96.02 | 17.36 | 13.56–21.15 |
|  | Yes | 16 | 3.98 | 43.75 | 19.34–68.16 |
| **PTE** | No | 384 | 95.52 | 16.41 | 12.69–20.13 |
|  | Yes | 18 | 4.48 | 61.11 | 38.48–83.73 |
| **Days in ICU** | ≤ 3 | 322 | 80.10 | 12.11 | 8.53–15.69 |
|  | > 3 | 80 | 19.90 | 43.75 | 32.83–54.65 |

Distribution of the study variables according to the crude and adjusted relative risks according to the Poisson regression model with robust variance and their respective 95% confidence intervals.

| **Variables** | **Relative Risk (RR)** | | **Relative Risk (RR) Adjusted*** | |
| --- | --- | --- | --- | --- |
|  | **RR (95 % IC)** | **p-value** | **RR (95% IC)** | **p-value** |
| **Block 1 – Demographic** |  |  |  |  |
| **Sex** |  | 0.4394 |  | 0.5128 |
| Women | 1 | - | 1 | - |
| Men | 1.18 (0.78–1.79) | 0.4394 | 1.14 (0.77–1.69) | 0.5128 |
| **Age** |  | < 0.0001 |  | < 00001 |
| 60 - 79 | 1 | - | 1 | - |
| 80 - 89 | 3.52 (2.00–6.17) | < 0.0001 | 350 (1.99–6.15) | < 0.0001 |
| > 90 | 6.13 (3.45–10.90) | < 0.0001 | 6.11 (3.44–10.87) | < 0.0001 |
| **Block 2 – Preoperative** |  |  |  |  |
| **Hemoglobin** |  |  |  | 0.0334 |
| ≤ 10 | 1.85 (1.21–2.81) | 0.0041 | 1.54 (1.03–2.29) | 0.0334 |
| > 10 | 1 | - | 1 | - |
| **SAH** |  | 0.0901 |  | 0.2475 |
| No | 1 | - | 1 | - |
| Yes | 1.64 (0.92–2.92) | 0.0901 | 1.39 (0.79–2.45) | 0.2475 |
| **DM** |  | 0.1412 |  | 0.6915 |
| No | 1 | - | 1 | - |
| Yes | 1.36 (0.90–2.06) | 0.1412 | 0.92 (0.63–1.36) | 0.6915 |
| **Neurological disorders** |  | 0.0267 |  | 0.5050 |
| No | 1 | - | 1 | - |
| Yes | 1.60 (1.06–2.44) | 0.0267 | 1.14 (0.77–1.69) | 0.5050 |
| **COPD** |  | < 0.0001 |  | 0.0002 |
| No | 1 | - | 1 | - |
| Yes | 3.04 (1.97–4.69) | < 0.0001 | 2.39 (1.52–3.78) | 0.0002 |
| **Surgical risk** |  | < 0.0001 |  | < 0.0001 |
| Low to moderate | 1 | - | 1 | - |
| High | 4.43 (2.84–693) | <0.0001 | 3.18 (2.00–5.06) | < 0.0001 |
| **Femur fracture** |  | 0.8222 |  | 0.1681 |
| Intracapsular | 1 | - | 1 | - |
| Extracapsular | 0.95 (0.63–1.44) | 0.8222 | 1.32 (0.89–1.97) | 0.1681 |
| **Block 3 – Postoperative** |  |  |  |  |
| **Type of surgery** |  | < 0.0001 |  | 0.0003 |
| Non-surgical | 3.47 (2.01–5.99) | < 0.0001 | 2.04 (1.31–3.16) | 0.0003 |
| Osteosynthesis | 1.27 (0.69–2.32) | 0.4431 | 1.03 (0.64–1.64) | 0.9108 |
| Arthroplasty | 1 | - |  |  |
| **Respiratory infection** |  | < 0.0001 |  | < 0.0001 |
| No | 1 | - | 1 | - |
| Yes | 15.75 (9.26–26.77) | < 0.0001 | 7.27 (3.98–3.26) | < 0.0001 |
| **Urinary tract infection** |  | < 0.0001 |  | < 0.0001 |
| No | 1 | - | 1 | - |
| Yes | 2.86 (1.93–4.25) | < 0.0001 | 2.04 (1.44–2.89) | < 0.0001 |
| **Other infection** |  | 0.0024 |  | 0.0251 |
| No | 1 | - | 1 | - |
| Yes | 2.52 (1.39–4.58) | 0.0024 | 1,98 (1.09–3.62) | 0.0251 |
| **PTE** |  | < 0.0001 |  | 0.0202 |
| No | 1 | - | 1 | - |
| Yes | 3.72 (2.42–5.74) | < 0.0001 | 1.98 (1.11–3.52) | 0.0202 |
| **Days in ICU** |  | < 0.0001 |  | 0.1683 |
| ≤ 3 | 1 | - | 1 | - |
| > 3 | 3.61 (2.46–5.31) | < 0.0001 | 1.27 (0.90–1.80) | 0.1683 |

* Prevalence Ratio adjusted by the variables of the Log-Binomial regression model: age, surgeries performed, infection and days in ICU

**Results**

In the bivariate analysis, a statistically significant association was observed between the mortality outcome and the following variables: age 80 to 89 (RR = 3.52; 95% CI 2.00 - 6.17) age > 90 (RR = 6.13; 95% CI 3.45 - 10.90), hemoglobin ≤ 10 (RR = 1.85; 95% CI 1.21 - 2.81), neurological disorder (RR = 1.60; 95% CI: 1.06 - 2.44), COPD (RR = 3.04, 95% CI: 1.97 - 4.69), high surgical risk (RR = 4.43, 95% CI: 2.84 - 6.93), non-surgical (RR = 3.47, 95% CI: 2.01 - 5.99), respiratory infection (RR = 15.75, 95% CI: 9.26 - 26.77), urinary infection (RR =2.86, 95% CI: 1.93 - 4.25), other infections (RR = 2.52, 95% CI: 1.39 - 4.58), PTE (RR = 3.72, 95% CI: 2.42 - 5.74), more than three days in ICU (RR = 3.61, 95% CI: 5.31).

The tolerance indicator for multicollinearity ranged from 0.60 to 0.95, indicating no strong multicollinearity between the independent variables within each stage.

In the second stage, age was included along with the block of in-hospital variables, and only the variables hemoglobin ≤ 10 (PR = 1.54; 95% CI 1.03–2.29), COPD (PR = 2.39; 95% CI 1.52–3.78), and high surgical risk (PR = 3.18; 95% CI 2.00–5.16) showed a significant association with mortality, even after adjustment. Therefore, these variables were maintained for the next block analysis.

In the second stage of the multiple Poisson regression analysis, age was included with the in-hospital variables. Only the variables hemoglobin ≤ 10, COPD, and high surgical risk were significantly associated with mortality outcome. After adjustment for the variables mentioned above, hemoglobin ≤ 10 decreased in the association (PR = 1.54; 95% CI: 1.03; 2.29), COPD decreased in the association (PR = 2.39; 95% CI: 1.52; 3.78) and the high surgical risk decreased in the association (PR = 3.18; 95% CI: 2.00; 5.16) all in relation to bivariate analysis. The variables age, hemoglobin, COPD, and surgical risk were maintained to analyze the next block.

In the last stage, age, hemoglobin, COPD, and high surgical risk were included with the postoperative variables. After adjustment for possible confounders, the variables occurrence of respiratory infection, urinary tract infection, other infections, and PTE were significantly associated with mortality outcome. After adjusting for the variables mentioned above, respiratory infection decreased in the association (PR = 7.27; 95% CI: 3.98; 13.26), urinary infection decreased in the association (PR = 2.04; 95% CI: 1.44; 2.89), other infections decreased in the association (PR = 1.98; 95% CI: 1.09; 3.62), and PTE decreased in the association (PR = 1.98; 95% CI: 1.11; 3.52) all in relation to the bivariate analysis.

**References**

- - - 1. Guangyong Zou. A Modified Poisson Regression Approach to Prospective Studies with Binary Data. Am J Epidemiol 2004;159:702-706.
      2. Victora CG, Huttly SR, Fuchs SC, Olinto MT. The role of conceptual frameworks in epidemiological analysis: a hierarchical approach. Int J Epidemiol*.* 1997;26(1):224-227.

doi: 10.1093/ije/26.1.224
