# Supplementary material for: Osteoporotic hip fracture—Comorbidities and factors associated with in-hospital mortality in the elderly: A nine-year cohort study in Brazil
Source: PLoS One. 2022 Aug 12;17(8):e0272006. doi: 10.1371/journal.pone.0272006 (PMC9374234; doi:10.1371/journal.pone.0272006)
Supplement: Attachment — Submitted filename: Response to reviewers.docx [file pone.0272006.s003.docx]

Regarding the data availability

Response: We have included two Supporting information files (S1 and S2) to support our data and added the paragraph below.

"For identification and description of the variables, the data were collected through the patients' admission records in the Orthopedics ward in the mentioned period (2010-2018) through Trackcare@ electronic medical records. After that, each clinical record related to the identified patient was analyzed individually and sequentially in the daily record of the nursing team for data conference, where the selection of the variables of interest to the study and transcription to a database (Excell@) for further statistical analysis (S1)."

----------------------------------------------------------------------------------------------------------

Reviewer #1: the authors have captured 400 hip fracture patients over 9 years and characterized the factors that influenced mortality. Their results are consistent with a number of other similar publications. This is unique in that it addressed Brazil medicine.

1. There are some key concerns with the patient population. This study enters less than one patient per week for hip fractures. The usual general hospital that handles hip fractures sees one per day. Were the 400 patients all the hip fractures at that hospital. If no how were they chosen and was there potential bias. If these are all the patients then the hospital is weak in hip fracture experience and why so few? Where did these patients come from?

Response: Regarding the number of fractures selected for the study, we clarify the questioned points: The research site was in an Orthopedics and Traumatology service of a Hospital in the Brazilian Public Health System with approximately 90,000 consultations. In a developing country like Brazil, the proportion of elderly patients in this population is less than 10%. The monthly statistic for orthopedic patients is around 15,000 consultations, and on average, about 1500 surgeries are performed monthly. However, they are general care for several orthopedic pathologies, traumatic or elective. This scenery is why this hospital is accredited by the Brazilian Society of Orthopedics and Traumatology to train resident physicians. The selection of patients for the study strictly followed the inclusion criteria: elderly patients (in Brazil, over 60 years old) with femur fractures caused by falling from their own height, i.e., it should be specified in the medical record that it was a low-impact trauma. This explains the lower number of patients in the sample among the general population and attendances. One of the reasons for the extension of time to increase the number of patients in the sample was to minimize biases. We did not include data from patients who had doubts about the type of fracture or missing data at the first visit, making it impossible to analyze the cohort with all the selected variables. The authors opted for this option when comparing the method with all the best studies already published on the subject cited in the manuscript's references, always trying to achieve the ideal reproducibility and a real analysis of the causes involved in mortality.

2. Also of concern is the long hospital stay. Around the world most hip fracture patients leave after 4-5 days going either home or to a rehab hospital. How do the authors explain this markedly long hospital stay. Are the patients both acute and then subacute?? Time to surgery has consistently been demonstrated to influence mortality. Why not in this group?? It is recommended that the patients should undergo surgery ideally within 24 hours of admission and certainly within the first 48 hours. How did this hospital rate and did the patients who were operated within 24 hours do compared to the others?

Response: The reviewer's extremely pertinent observations allow us to discuss the subject more deeply. As recorded along the manuscript, the objective of our study was to contribute to the evidence regarding a real analysis of the morbidity and mortality causes of the Brazilian elderly population due to osteoporotic fractures. There are three observations: 1) The sicker profile of the elderly population aging in Brazil. The elderly fell and fractured because they were sick, requiring more time after hospital admission to treat the underlying pathology—the reason for not performing interventional surgery in the first hours, as recommended. The tables demonstrate the higher number of patients with high surgical risk. The reason is that population aging in Brazil is recent, having started around the 1960s. However, life expectancy, which was around 60 years, increased to 80 years, without social preparation in several aspects, both in terms of individual and collective health quality. Some epidemiological studies performed with large samples of the Brazilian population report that 70% over 60 years have one or more comorbidities. Therefore, a relationship between the aging population of developing countries and the population's mortality was analyzed in this study. We reinforce that this study will serve as a model for other populations in developing countries like Brazil. 2) The few Brazilian studies performed on the subject do not have an analytical method, only a temporal descriptive with a smaller number of patients. Thus, although they describe the intervention time, it is impossible to affirm the relationship with mortality, as we did in our study after statistical analysis with significance. The authors conducted another study in Brazil that may confirm the longer intervention time, longer hospital stay and mortality in Brazilian public health services. (Indicators of morbidity and mortality by femur fractures in older people: A decade-long study in Brazilian hospitals. DOI:10.1590/1413-785220202803228393) In truth, with the publication, we aim to contribute to an expanded and detailed analysis of the subject. 3) The intervention sample before 24 hours was very small, so it was not possible to compare the groups.

3. Most of the medical issues are covered by the Charlson index and most of the OR risk is covered by the ASA score. Please provide them for this patient population this study has a very high mortality rate. Give us the 3-day rate rather than the 22-day rate.

Response: The choice of the score used to evaluate the surgical risk in the hospital is the Detsky index. The choice does not invalidate the evaluation because no score stands out regarding the accuracy in relation to the others. Despite several perioperative cardiac risk scales, little can be inferred about which has greater accuracy and their performance in different populations. A brief history is shown in the manuscript (please see below) that justifies the option of using the Detsky index in comparison to the others, in the advantage verified in the greater multiparametric evaluation giving greater reliability to the analyses in this population with multiple comorbidities, especially cardiovascular, as shown in the Brazilian epidemiology. The Charlson Comorbidity Index is a system for assessing life expectancy and includes diseases such as solid neoplasms and lymphomas. According to the study's inclusion and exclusion criteria, the patients were EXCLUDED because they are causes of pathological fractures. Everything was discussed with the team to have the necessary alignment and consistency to better analyze mortality in osteoporotic fractures in the general elderly population. The American Society of Anesthesiologists (ASA) Physical Status Classification System, introduced by Saklad et al. in 1941 and modified in 1963, classifies patients according to their general clinical status, based only on the presence or absence of systemic disease, into six possible physical levels, with increasing mortality percentage among the classes. It is easily applicable and widely used in preoperative evaluation, but it is quite generic. Algorithms for preoperative cardiac evaluation, such as the cardiac risk index described by Goldman et al. in 1977, the first multifactorial model specific for perioperative cardiac complications, and Detsky et al. in 1986 who added to Goldman's original model the evaluation of the presence of angina, previous history of myocardial infarction, or acute pulmonary edema, are closer to the evaluation of the aging Brazilian population reality. Therefore, a safe and broad index with a greater approach including cardiac parameters and confirming the correlation with mortality.

Manuscript:

Preoperative cardiac risk was analyzed using the Detsky Modified Cardiac Risk Index, 1986 [47], and patients classified as high surgical risk (RR 10.6) were significantly associated with the highest risk of death in our study. This assessment can be used to estimate possible risks resulting from the surgical procedure in each patient and, if possible, conducts to minimize these risks. This estimate is essential to provide the surgeon/team and patient/family with information that must be taken into account when comparing the procedure's possible benefits and harms in each case [48].

The association between atherosclerotic cardiovascular diseases and osteoporosis emphasizes epidemiological and physiopathogenic similarities in arterial wall calcification and osteogenesis considering the low bone mass, osteoporosis fractures, vascular calcification, extension of coronary and abdominal aorta injury, and cardiovascular mortality, regardless of age. Osteopenia and osteoporosis in the femoral neck were associated with a higher risk of severe coronary lesions [49, 50], and the reverse [50].

However, the Detsky Modified Cardiac Risk Index, 1986 [47] does not consider the SAH, present in 75% of patients in our study, in its score. Although this variable was not associated with a direct risk of death, patients with SAH had a higher mortality.

The high prevalence of cardiovascular diseases found in the elderly population in Brazil [51] raises two issues: 1) the established relationship between cardiovascular diseases and osteoporosis [52], and 2) SAH as an independent risk factor for increased mortality [53] to be considered, requiring evaluation regarding its prevention and associated risk factors.

----------------------------------------------------------------------------------------------------------

Reviewer #2: This is a retrospective multivariate analysis from Brazil of 402 patients with hip fractures over eight years to examine factors associated with in hospital mortality. The in-hospital mortality was 18% and not surprisingly older patients, those with comorbidities, those selected for non-operative treatment, and who develop infections were at risk for higher mortality.

The study was very comprehensive and its analysis of important variables and include some relatively novel methods of stratifying risk such as cardiac, pulmonary embolism etc. with quantitative scales. The Multi variable analysis was extremely well done and eloquently explained in the results. The results and conclusions are justified by their data and analytic methods. The manuscript was extremely well written and a pleasure to read.

1. A number weaknesses need to be taken into consideration when interpreting those data. First I do not think the ROC analysis on length of stay is compelling or generalizable and depends on local conditions. I do not believe it is useful and would be best deleted from the study. The selection bias regarding surgical versus non-surgical treatment is problematic and clearly results in the patients treated nonoperatively having higher mortality. One approach might be the patient altogether and only discussing hospital mortality of the operative patients or do this separately. The follow up is very short and only regarding inpatient mortality which may not generalize well to the rest of the world given the prolong length of stay seen in Brazil.

Response: The reviewer's extremely pertinent observations allow us to discuss the subject more deeply. As shown in several places in the manuscript, the objective of this work was to contribute to the medicine of developing countries, mainly regarding evidence in front of a real analysis of the morbidity and mortality causes of the Brazilian elderly population due to osteoporotic fractures. Please see below the discussion section:

"Surgery should be the most appropriate option for most patients [59], as it is associated with a shorter hospital stay and better rehabilitation [60]. Non-surgical interventions are reserved for patients with a severe debilitation, unstable patients with incurable severe diseases, or patients with terminal illnesses in the final stages of life [61]. Stable impacted fractures can also be considered for non-surgical treatment [62]. However, with conservative treatment, rehabilitation will probably be slower and limb deformity, more common [63]."

In this discussion, we tried to explain a hypothesis concisely for the sick profile of the aging population in Brazil and the surgical decision facing a fracture. In other words, the elderly who are ill have a greater chance of falling and, consequently, a fracture. Therefore, after hospital admission, they will need more time for the treatment and clinical stabilization of comorbidities, the reason for not performing surgery in the first hours, as recommended, and for possible non-surgical intervention. We chose to compare the groups because this is relevant information to public health, which will also help other developing countries deepen studies on the subject and contribute to the literature worldwide, bringing the fracture and the time variable several correlated factors.

2. 101 It seems like the aims should be stated in the introduction not the conclusion. Also, in the abstract you did not comment on the results regarding infections.

Response: Introduction: The aim of the study was to identify factors associated with in-hospital mortality among patients undergoing surgery or conservative treatment in an elderly population with multiple comorbidities treated in a public hospital in Brazil, a developing country, with a rapid population aging process.

The observation of the study's objective at the end was to reinforce the objectives of the analysis, with many possible variables and questions that could lead to confounding. Therefore, the aim of the study was not to compare independent variables with each other, but the association of comorbidities leading to the development of infections directly linked to mortality is clear.

3. 145 What was the time length of follow-up? Is this only in hospital mortality?

Response: Yes. The data collection was over nine years, but the data was collected from admission to outcome (discharge or mortality). We clarify the study's title: Osteoporotic hip fracture – comorbidities and factors associated with in-hospital mortality in the elderly: a nine-year cohort study in Brazil.

4. 149 you have a very good list of comorbidities however the cognitive state of the patient either acute or chronic are important predictors of outcome, risk of complications, mortality, and are perhaps the most important aspect regarding surgical decision making.

Response: Excellent observation and thank you for the question. We agree, the patient's clinical condition at the time for surgical approach should be paramount. In our study, the cognitive deficit was addressed as memory disorders or neurodegenerative dementia, such as Alzheimer's. But it was not the aim of this paper itself to go into detail, considering that we could incur in diagnostic biases. However, we emphasized the difference between altered state of consciousness, which would lead to the assumption of clinical emergencies, which does not fit in this case.

5. 199 the ROC analysis regarding length of stay is very dependent on the cultural and healthcare system utilized in Brazil and does not apply elsewhere. Also, significant bias in the patient to die early would have short length of stay but those who have prolong illness as long as he stays would have prolonged length of stay this would seem to confound this analysis. Please explain what postoperative time period means.

Response: Excellent question when referring to studies with the time variable. The surgical intervention was the cut-off point of the time analysis. Admission to surgery = preoperative. After surgery to the outcome (discharge or mortality) = postoperative. This period was statistically analyzed and represented in a curve graph with a point of inflection from the significant day for mortality incidence in relation to the mean. It reflects the importance of not only early intervention but also discharge with early rehabilitation. That was the goal, with emphasis on the time variable only. But we understand the observation. Chronic diseases can contribute both to the hypothesis of aggravation, which led to the fall from their own height, and therefore the health status at admission that does not allow early surgical intervention and in contributing to more complications after surgical stress. The study tried to minimize the bias of the time variable, considering the concomitance of other variables, separating this information and analysis into a specific topic and tables, and we consider important the permanence of the information. Most studies predominantly address the preoperative time. Our study showed the importance of comprehensive treatment in addressing the fracture, with rehabilitation and clinical care after the intervention. We assume that often after discharge, complications are not reported ( under-reporting). The extended in-hospital observation of this study was able to capture.

6. 305 Please explain what groups you were referring to.

Response Compare each time-related variable with the outcome mortality (yes or no) to evaluate the significance of the information (Table 4). However, we thank you for the analysis performed and will adjust this information in the manuscript.

"Considering the entire sample, patients who died had a longer hospital stay (31.28 ± 23.24); however, when comparing each variable, related to time with mortality outcome (yes or no), the postoperative period (21.05 ± 21.71) and longer stay in ICU (10.65 ± 16.62) presented a significant association with mortality (Table 4)."

7. 324 Need to define whether this is total hip arthroplasty or hemi arthroplasty.

Response: In this hospital, total hip arthroplasty is predominantly performed. The inclusion criteria were respected, and emphasis was placed on selecting the patients for the study.

8. 325 One of the major weaknesses of the study is that you do not specify indications for osteosynthesis, arthroplasty or non-operative treatment. These are largely dependent fracture type as osteosynthesis would typically use for intertrochanteric, subtrochanteric , and some femoral neck fractures where is hemiarthroplasty for displaced femoral neck fractures. Since these cannot be used interchangeably it makes no sense comparing these surgeries. Further non operative care is typically selected for those with greater trochanteric fractures or in patients too sick to have surgery or who have a very short life expectancy. Thus, significant selection bias is present which accounts for the higher mortality.

Response: We would like to thank you again for thoroughly and carefully reading our study that took nine years of data collection. We have tried to keep up with the best studies published on the subject worldwide. However, because we have covered many factors, this relevant information, discussed several times among the authors, was not explicitly included in the discussions because it is considered widely known. Instead, we cite in the discussion and paste the references to deepen the theme by addressing complementary points to this information, such as age influencing the bone quality and consequently the fracture mechanism and subsequent surgical decision.

"Considering the increase in life expectancy worldwide, especially in developing countries [33], the association among longevity, osteoporotic hip fractures, and mortality becomes relevant [28], raising the question of how these injuries affect health systems [34]. The age group over 90 years, which was identified by the hierarchical model of multiple regression as a factor associated with a six-fold higher RR for death [RR 6.11 (3.44–10.87)], relates the factors attributed to the senescence of this age group [35] both with bone degeneration, which accounts for the highest incidence of fracture mechanisms [36], and with a higher risk of fracture complications [37]."

"Regarding the hip fracture trauma mechanism pattern, these are classified by their site as an evidence of prognostic implications [58]. Surgery should be the most appropriate option for most patients [59], as it is associated with a shorter hospital stay and better rehabilitation [60]. Non-surgical interventions are reserved for patients with a severe debilitation, unstable patients with incurable severe diseases, or patients with terminal illnesses in the final stages of life [61]. Stable impacted fractures can also be considered for non-surgical treatment [62]. However, with conservative treatment, rehabilitation will probably be slower and limb deformity, more common [63]."

9. 463 I think you need to significantly expand study limitations particular problem of selection bias, unknown indications for surgery, not including cognitive state patient, only including in-hospital mortality.

We tried to reference relevant studies conducted to provide the reader with the necessary information to complement the limitations of the study in question.

Response: "The challenge of establishing a pattern among the several variables associated with worsening and to mortality outcomes [25] is related to the fact that these associations, as well as the mortality rate assessments reported in different studies involving different populations and methodologies, also present regional variations [16, 17, 26-28]."
