# Supplementary material for: Osteoporotic hip fracture—Comorbidities and factors associated with in-hospital mortality in the elderly: A nine-year cohort study in Brazil
Source: PLoS One. 2022 Aug 12;17(8):e0272006. doi: 10.1371/journal.pone.0272006 (PMC9374234; doi:10.1371/journal.pone.0272006)
Supplement: Attachment — Submitted filename: Response to reviewers.docx [file pone.0272006.s004.docx]

Reference list

We have included two new references

Maley JH, Wanis KN, Young JG, et al Mortality forecasts models, causal effects, and end-of-life decision making in the intensive care unit. BMJ Health & Care Informatics 2020; 27:e100220. doi: 10.1136/bmjhci-2020-100220.

Peterle VCU, Geber Junior JC, Darwin Junior W, Lima AV, Bezerra Junior PE, Novaes MRCG. Indicators of morbidity and mortality by femur fractures in older people: a decade-long study in Brazilian hospitals. Acta Ortop Bras. 2020;28(3):142-148. doi.org/10.1590/1413-785220202803228393.

Reviewer #1: The authors have stressed the underdeveloped nature of their country and feel that they should not be held to the highest level of care. There is a large number of patients that are not operated in a timely fashion. If we accept this concept of a developing nation, then the article is clear and well defined within their capacity. The only point would be in the abstract to clearly identify this limitation to the readers.

Response: We have made some changes along the text in the abstract, introduction (123-127), discussion (350-354, 364-369, 473-501), and conclusion (518-529) sections.

Reviewer 1 has asked that you add a brief discussion about the limitations of care in your setting, relative to best practices in wealthy countries.  This is a good idea and should be done.

Response: The extremely pertinent observations allow us to discuss the subject in greater depth. As suggested in the review process, we registered in several places in the manuscript that the objective of this study is to contribute to the medicine of developing countries, such as Brazil, being the largest study performed so far on the subject in the country, with an adequate methodology to produce evidence for a real analysis of the causes of morbidity and mortality of the elderly population due to osteoporotic fractures.

Currently, the main references on the subject are almost exclusively from developed countries, with healthcare organization systems managed by healthcare operators, electronic medical records using digital technologies (Machine Learning), databases (Big Data), or artificial intelligence, which provide advanced methods to capture, store, and analyze patient data, and which allow almost real time analysis on a research question, generating evidence for decision making that is followed all over the world.

In Brasil, patient care protocols are followed based on evidence generated in developed countries. However, professionals deal with dilemmas and anguish when facing the knowledge of the best evidence of intervention and try to apply them, but sometimes do not obtain the results expected and demonstrated in these studies.

When proposing this study, the authors gathered from the observation of real-life outcomes - discharge or death, the several hypotheses that originated the research questions, from the health conditions of the population assisted, or the course of fracture treatment from admission to the intervention, and studied a method of data collection and analysis development, regarding the variables of interest, that demonstrated significance for the construction of evidence.

The connection between our results and the international literature references was made at every point. The much-discussed point in the review regarding the low early intervention in this study was also honestly stated in the text.

There is no lack of knowledge of the evidence generated by the scientific community worldwide on this variable in the fracture intervention aspect. Still, we cannot extrapolate this description of early intervention in the population of this study. Since this is a cohort study, the conclusions were described based on the variables that showed association by statistical method.

We have tried within the limits of the method, to separate the variables related to the individual and the variables related to the health system in the care of these patients to as not to generate confusion and clarify the readers the main aspects that would be associated with the mortality outcome.

We suppose and dialog in the manuscript that chronic diseases can contribute to the hypothesis of aggravation leading to a fall from their height; therefore, the poor health status at admission does not allow early surgical intervention and contributes to more complications after surgical stress. In this population, most present a preoperative assessment with high cardiovascular risk, which we also have described so the reader can raise this hypothesis.

In other words, if the patient's previous health conditions at admission, such as multiple comorbidities, made surgery unfeasible, in case of no early intervention, considering the period of clinical stability for the patient to be fit for surgery. However, even this long period for intervention was no longer associated with mortality as postoperative period. We have addressed this matter as explicitly suggested in the manuscript review process.

Despite the Brazilian Unified Health System being universal and of unrestricted access, health interventions may be conditioned to the availability of resources, equipment, and management issues of the public system, which is a reason to question whether there were limitations in the management of the pathology. We described this observation in the manuscript.

In an analogy to Real World Evidence (RWE), the aim of the study was to generate evidence by filling gaps that clinical trials in other populations cannot complete in these populations.

Also, in analogy, the FRAX algorithm, developed by the Sheffield Bone Metabolic Diseases Centre, which calculates the probability of fracture from easily obtained clinical risk factors for women and men, could only be used in Brazil after the algorithm adaptation for the Brazilian population. Zerbini CAF et al. Incidence of hip fracture in Brazil and the development of a FRAX model. Archives of Osteoporosis (2015) 10; 28).

One look at the health conditions of the Brazilian elderly population with femur fracture due to bone fragility, dialoguing through knowledge of the best evidence, seeks to demonstrate through science, the importance of developing safe and optimized protocols for comprehensive treatment in the approach to fracture in developing countries.

Brazil has more than 500,000 physicians registered by the Federal Council of Medicine. However, according to the latest study of Medical Demography in Brazil, released in 2020, there are approximately 18,000 professionals in Orthopedics and Traumatology to serve a population of more than 280 million inhabitants. That is about six professionals for every 100,000 inhabitants.

In summary, as recorded in several places in the manuscript, the objective of this study was to contribute to a fundamental analysis of the causes of morbidity and mortality of the Brazilian elderly population due to osteoporotic fractures. We made two observations: 1) The more ill profile of the aging elderly population in Brazil. That is, the elderly fall and fracture because they were sick, requiring more time after hospital admission to treat the underlying pathology. Hence, as recommended, the reason for not performing interventional surgery in the first few hours. The tables illustrated in the manuscript show the higher number of patients with high surgical risk. This is explained because Brazil's population aging is recent, starting around the 1960s. However, life expectancy, which was around 60 years then, has increased to 80 years, without social preparation in several aspects, both in individual and collective health quality. Some epidemiological studies performed with considerable samples of the Brazilian population report that 70% of the population over 60 years has one or more comorbidities. Therefore, a relationship between the aging population of developing countries and the population's mortality was analyzed in this study. We reinforce that this study will serve as a model for other populations in developing countries like Brazil. The second point: 2) The few Brazilian studies performed on the subject do not have an analytical method, only a temporal descriptive with fewer patients. Thus, although they describe the intervention time, it is impossible to affirm the relationship with mortality, as we did in this study after statistical analysis with significance. The authors conducted another study in Brazil that may confirm the longer intervention, hospitalization time, and mortality in Brazilian public health services (DOI:10.1590/1413-785220202803228393). Reality that, with this publication, we seek to contribute to an expanded and detailed analysis of the subject.
